# Supplementary material for: Identification and functional analysis of non-coding regulatory small RNA FenSr3 in Bacillus amyloliquefaciens LPB-18
Source: PeerJ. 2023 May 15;11:e15236. doi: 10.7717/peerj.15236 (PMC10194069; doi:10.7717/peerj.15236)
Supplement: Supplemental Information 4 [file peerj-11-15236-s004.zip › KO/CK-vs-T1_map/map04140.html]

KEGG PATHWAY: Autophagy - animal - Reference pathway


|  |  |
| --- | --- |
| **Autophagy - animal - Reference pathway** |  |

[
Pathway menu
| Organism menu
| Pathway entry
| Show description
| User data mapping
]

Autophagy (or macroautophagy) is a cellular catabolic pathway involving in protein degradation, organelle turnover, and non-selective breakdown of cytoplasmic components, which is evolutionarily conserved among eukaryotes and exquisitely regulated. This progress initiates with production of the autophagosome, a double-membrane intracellular structure of reticular origin that engulfs cytoplasmic contents and ultimately fuses with lysosomes for cargo degradation. Autophagy is regulated in response to extra- or intracellular stress and signals such as starvation, growth factor deprivation and ER stress. Constitutive level of autophagy plays an important role in cellular homeostasis and maintains quality control of essential cellular components.

|  |  |  |
| --- | --- | --- |
| Reference pathway | 184% 150% 122% 100% 82% 67% 55% | 图片下载 |
